# Repeated Human Exposure to Semivolatile Organic Compounds by Inhalation: Novel Protocol for a Nonrandomized Study

**DOI:** 10.2196/51020

**Published:** 2023-10-13

**Authors:** Elena Reale, Nancy B Hopf, Florian Breider, Dominique Grandjean, Catherine Pirard, Corinne Charlier, Holger M Koch, Aurélie Berthet, Guillaume Suarez, Myriam Borgatta

**Affiliations:** 1 Department of Occupational Health Center for Primary Care and Public Health (Unisanté) University of Lausanne Lausanne Switzerland; 2 Swiss Centre for Applied Human Toxicology Basel Switzerland; 3 Central Environmental Laboratory Ecole Polytechnique Fédérale de Lausanne Lausanne Switzerland; 4 Center for Interdisciplinary Research on Medicines University of Liege Liege Belgium; 5 Laboratory of Clinical, Forensic and Environmental Toxicology University Hospital of Liege Liege Belgium; 6 Institute for Prevention and Occupational Medicine of the German Social Accident Insurance Institute of the Ruhr-University Bochum Bochum Germany

**Keywords:** DEHP, diethylhexyl phthalate, healthy participants, inhalation exposure, phthalates, portable aerosol-generating device, protocol, semi-volatile organic compounds, SVOCs, toxicokinetics study, toxicology

## Abstract

**Background:**

Semivolatile organic compounds (SVOCs) comprise several different chemical families used mainly as additives in many everyday products. SVOCs can be released into the air as aerosols and deposit on particulate matter during use by dispersion, evaporation, or abrasion. Phthalates are SVOCs of growing concern due to their endocrine-disrupting effects. Human data on the absorption, distribution, metabolism, and excretion (ADME) of these compounds upon inhalation are almost nonexistent.

**Objective:**

The goal of this study is to develop a method for repeated inhalation exposures to SVOCs to characterize their ADME in humans.

**Methods:**

We will use diethylhexyl phthalate (DEHP), a major indoor air pollutant, as a model SVOC in this novel protocol. The Swiss official Commission on Ethics in Human Research, Canton de Vaud, approved the study on October 14, 2020 (project-ID 2020-01095). Participants (n=10) will be repeatedly exposed (2 short daily exposures over 4 days) to isotope-labeled DEHP (DEHP-d4) to distinguish administered exposures from background exposures. DEHP-d4 aerosols will be generated with a small, portable, aerosol-generating device. Participants will inhale DEHP-d4-containing aerosols themselves with this device at home. Air concentrations of the airborne phthalates will be less than or equal to their occupational exposure limit (OEL). DEHP-d4 and its metabolites will be quantified in urine and blood before, during, and after exposure.

**Results:**

Our developed device can generate DEHP-d4 aerosols with diameters of 2.5 μm or smaller and a mean DEHP-d4 mass of 1.4 (SD 0.2) μg per puff (n=6). As of May 2023, we have enrolled 5 participants.

**Conclusions:**

The portable device can be used to generate phthalate aerosols for repeated exposure in human studies.

**International Registered Report Identifier (IRRID):**

DERR1-10.2196/51020

## Introduction

The general population is exposed every day to semivolatile organic compounds (SVOCs) through inhalation [1-4], but few data exist on the internal (eg, blood) concentrations of these compounds when inhaled. One reason for this lack of data is the absence of a device to aerosolize SVOCs for controlled human exposure over several days. These compounds cannot be tested in common devices used for volatile chemicals such as solvents in whole-body exposure chambers. SVOCs are found in the air as liquid aerosols and deposited onto solid particles that are easily resuspended in the air and directly available through inhalation [5]. They are used as active ingredients in pesticides, cleaning agents, and personal care products, as well as additives in materials such as plastics, floor coverings, furnishings, and electronic components [4]. More than a thousand SVOCs are high-production-volume chemicals (HPVCs) [6]. Many of these have been associated with adverse health effects and have since been removed from commercial use [7,8]. Some are presumed (category 1B) endocrine-disrupting chemicals (EDCs) [9,10], including, among others, phthalates [11-14], brominated flame retardants [15], bisphenol A [16], nonylphenol [17], and some pesticides [18]. SVOCs are found in higher abundance indoors than outdoors [19,20] and are detected in both blood and urine collected from the general population [21]. This confirms the omnipresent and concurrent body burden.

**Figure 1:**
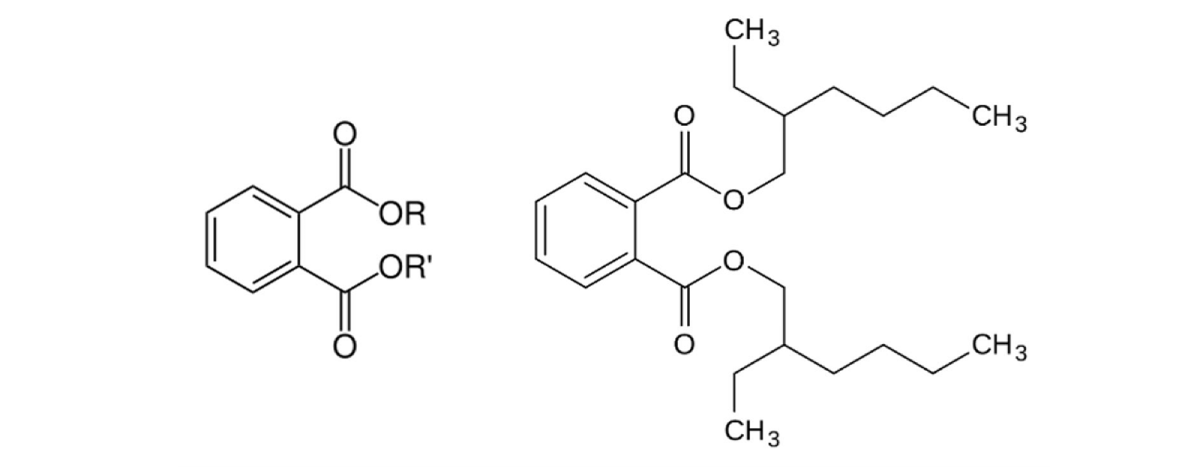
Generic chemical structure of phthalates, where R and R’ are alkyl or aryl moieties (left), and chemical structure of diethylhexyl phthalate (DEHP) (right).

Phthalates are a specific family of SVOCs used to impart flexibility to polymer plastics (ie, plasticizers) [22]. Phthalates are not covalently bound to the polymer matrix. Therefore, they can migrate to the surface and be slowly released into the air [21-24]. Phthalates with short alkyl chains, such as diethyl phthalate (DEP), have the highest vapor pressures and can also be released in the air in the vapor phase. Phthalates with long alkyl chains, such as diethylhexyl phthalate (DEHP), have lower vapor pressures and tend to adsorb to aerosol particles or dust [23] (Figure 1). DEHP has been reported to be the most abundant phthalate in house dust, with measured concentrations up to 5.3 mg/g [24,25].

Children and adults living in industrialized countries are estimated to spend around 17-19 hours and 21 hours per day indoors (ie, at home and in the working environment), respectively [26]. There is considerable concern regarding phthalate exposures because several epidemiological studies have shown associations between exposures to some phthalates and retarded male reproductive development [11,12], altered semen quality [13,14], endometriosis [27,28], and respiratory diseases in children [29]. When inhaled, phthalates can reach the alveoli, be absorbed into the blood, and then be distributed to potential target tissues, where they may trigger an effect. Therefore, absorption rates (ie, rate at which the molecule enters blood), internal doses (ie, parent compound concentrations in blood), as well as metabolic and elimination rates (ie, rate of metabolites’ formation and elimination, usually in urine), are important toxicokinetic parameters to accurately determine the total exposures in the human body and, thus, potential risks to the population. Although urinary metabolite ratios were reported to be different between humans and animals [30-34], these toxicokinetic parameters have not been studied in humans exposed to phthalates through inhalation as a single exposure route. The few human toxicokinetic studies conducted with phthalates were mainly after ingestion [35-38]. A total of 2 human studies have been conducted on phthalates, including simultaneous exposure through the skin and inhalation routes [39,40]. These studies only provided estimated toxicokinetics (absorption, distribution, metabolism, and excretion [ADME]) for the inhalation route. Since inhalation is considered a significant indoor route of exposure for some phthalates [41], toxicological studies need to be performed to understand phthalates’ ADME through this exposure route. However, a procedure to expose healthy participants to SVOCs is lacking due to the absence of devices for human studies with SVOCs through inhalation. We aim to fill these gaps by describing the required steps to obtain human ADME data after inhalation. The SVOC selected in this study is DEHP, a model SVOC due to its major presence in the indoor environment (air and dust) and human populations (urine). This study is divided into 2 parts. Part I describes the dosing procedure and how to generate aerosols at environmentally relevant phthalate concentrations with a device suitable for chronic inhalation exposure. Airborne phthalate aerosols will be produced based on the technology of commercially available “electronic nicotine delivery systems” (ENDS), which have proven efficacy in generating aerosols for human use [42,43]. ENDS are generally used to generate nicotine or flavored aerosols from highly viscous liquids such as glycerol (viscosity=1412 mPa·seconds) making this technology suitable for viscous chemicals such as some phthalates. Part II provides the study protocol for participants exposed to ring-deuterated DEHP for several days. Unisanté has strong expertise in toxicological studies with healthy participants and has adapted its protocols for repeated, at-home exposure. We will show the results of the first participant here as proof of concept.

## Methods

### Part I: Dosing Protocol

#### Portable Dosing Device

The commercial ENDS “Endura T20-S” (Innokin) was chosen as the portable dosing device for participants’ exposure to DEHP. ENDS consists of a battery-powered heated coil that functions as a thermal atomizer, a mouthpiece, and an e-liquid tank. Generally, the e-liquid is mainly composed of nicotine, flavoring agents, and ethanol in a base of propylene glycol (PG) and vegetable glycerol (VG). Base composition can vary from a PG/VG ratio of 20/80 to 70/30 and is typically 50/50. When the user activates the heated coil by pressing a button on the ENDS, the e-liquid is vaporized, releasing a flavored nicotine-containing aerosol called a “puff.” The user inhales this aerosol through the mouthpiece.

#### E-Liquids

Aerosols generated by ENDS are complex mixtures containing volatile and semivolatile gases (mainly PG and glycerol) and nonvolatile particles (mainly glycerol, PG, and nicotine) [44,45]. The e-liquid formulated in this study is composed of glycerol (purity 99.5%, Hänseler Swiss Pharma), PG (PG 0 mg/mL nicotine, Revolute), ethanol (absolute for analysis EMSURE, Supelco, Merck), and DEHP. Ring-deuterated DEHP (DEHP-d4, CAS# 93951-87-2, Sigma Aldrich) is used to differentiate exposures generated in our experiment to environmental and laboratory DEHP contaminations.

#### Aerosol Generation and Vaping Machine

**Figure 2:**
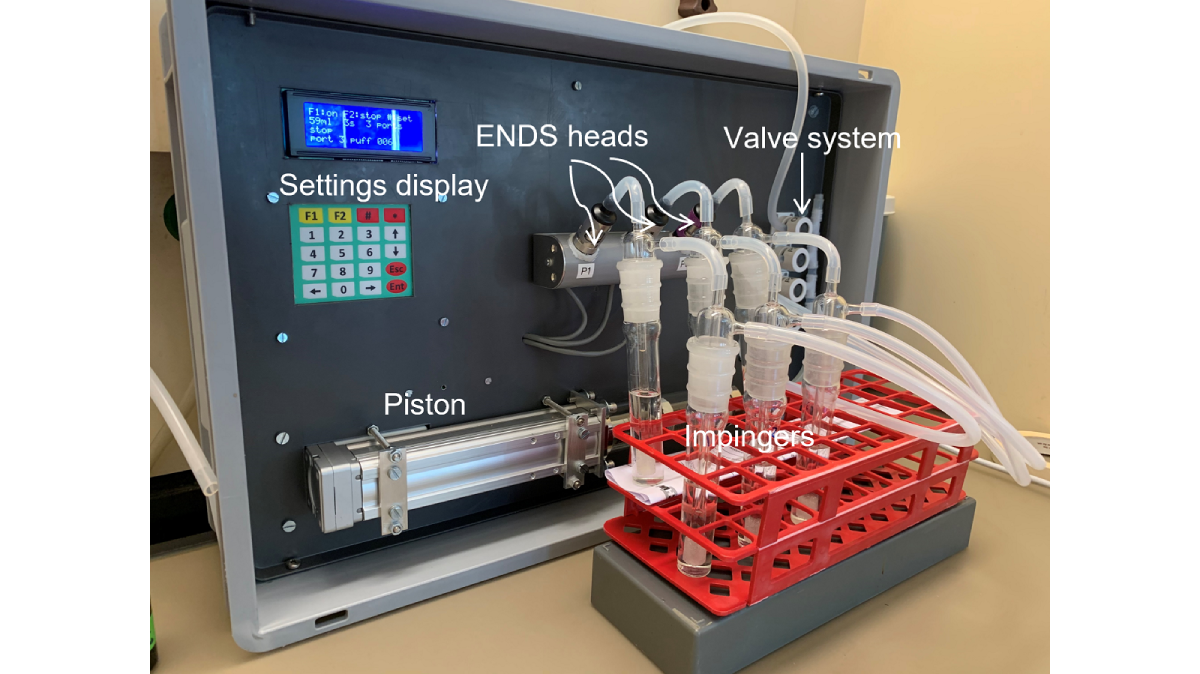
Aerosol generation tests with our vaping machine. A total of 3 electronic nicotine delivery systems (ENDS) heads were directly screwed onto the vaping machine. Each ENDS head was attached to 2 sampling bottles (impingers) in series by silicon tubes. The impingers were attached to the valve system of the vaping machine and could be set to a specific vaping volume, duration, and frequency through the display at the top left.

To adapt the ENDS to generate DEHP aerosols at controlled concentrations, we used a vaping machine that was developed in the Unisanté laboratory in collaboration with the University Centre of Legal Medicine of Lausanne (CURML) [46]. The purpose of the vaping machine was to replicate the puffing behavior of an individual using an ENDS according to the method n°81 recommended by the Cooperation Centre for Scientific Research Relative to Tobacco (CORESTA) [47]. This method describes the requirements for the generation and collection of e-cigarette aerosols for analytical testing purposes and led to an ISO (International Organization for Standardization) standard (20768:2018). Our vaping machine included 3 ports, each for an ENDS head (e-liquid tank, atomizer, and mouthpiece), a power supply, a piston syringe, a pinch-valve system, and silicon tubing (Figure 2). The machine was equipped with its own power supply, which replaced the individual ENDS batteries. This provided a steady-state power of 12 W throughout all experiments. The replacement of the ENDS battery by the machine’s power supply precisely controlled puff duration during the experiments. The piston, in combination with the pinch valve system, controlled the puff volume, that is, the volume leaving the ENDS. Puff volumes were trapped by n-hexane in subsequent glass impingers (with P1 porosity fritted nozzle tip, joint connection NS 29/32, Bistabil, Sigma Aldrich) for analytical analysis. Puff duration (3 seconds per puff), volume (55 mL per puff), and frequency (30 second between puffs) were set up as per CORESTA criteria. Tests were carried out in triplicates. Control tests served to verify that the entire setup was not contaminated by DEHP-d4. A total of 3 ENDS with 0.8-ohm atomizers were used for all the experiments. New atomizers were used in each experiment.

#### E-Liquid Formulation and Testing Conditions Optimization

A first series of tests was carried out on the vaping machine to optimize the e-liquid formulation for generating stable aerosol concentrations as well as trapping conditions. We prepared several formulations with different PG/VG ratios (from 0/100 to 50/50), different ethanol amounts (0%-10%), and different DEHP amounts (0.002%-0.3%, w/w) to determine e-liquid formulations that allowed DEHP to be dissolved in the e-liquid and aerosolized by the atomizer. For each ENDS, DEHP-d4 was quantified in the n-hexane of all impingers. The number of impingers (2 and 3), impinger volume (20 and 100 mL), and n-hexane volume in each impinger (13, 50, 75, and 80 mL) were tested for optimal DEHP-d4 trapping conditions.

#### DEHP-d4 Mass Balance and Aerosols Concentrations

We carried out a second series of experiments with the vaping machine to determine mass balance and confirm DEHP-d4 concentration in the aerosols. DEHP-d4 was quantified in the n-hexane of all impingers, in the e-liquid before and after the experiment, and in an acetone/ethanol (50/50 v/v) mixture (acetone, suitable for high-pressure liquid chromatography [HPLC] ≥99.9%, Sigma Aldrich) that was used to extract DEHP-d4 from disassembled ENDS parts at the end of the experiment by the Central Environmental Laboratory of the Ecole Polytechnique Fédérale of Lausanne (EPFL).

#### DEHP-d4 Stability in E-Liquid

The stability of the DEHP-d4 concentration in e-liquid was an important control step for the participants’ safety and to avoid any exposure concentration variability between the participants. The stability test for DEHP-d4 in the e-liquid was performed on the e-liquid stored at room temperature for 1 week and frozen at –20 °C for 2 weeks.

#### Aerosol Particle Mass Distribution in E-Liquid

A portable optical particle counter (aerosol spectrometer, model 1.109, GRIMM) was used to determine the mass distribution of e-liquid particles in the aerosols and assess deposition in the respiratory system [48,49]. Aerosols were generated by equipping the vaping machine with ENDS. The e-liquid used for these tests was composed of a PG/VG ratio of 50/50 with 1% ethanol and was not spiked with DEHP-d4. A total of 6 puffs of aerosol (55 mL×6=330 mL) were diluted in ambient air in a 3 L sampling bag (Tedlar sampling bag, Nutech Instruments Inc). The dilution in ambient air simulated the aerosol dilution in inhaled air by an individual puffing, assuming a 55 mL puff and a 500 mL tidal volume (330 mL/3000 mL=55 mL/500 mL). The particle size distribution profile was then measured. A control test was performed with ambient air.

#### DEHP-d4 Exposure Concentration

The concentration of DEHP-d4 in the e-liquid (percentage by weight) was set at a value that produced DEHP-d4 concentrations for participant exposures (C_exp_, mg/m^3^) at or below the Swiss occupational exposure limit (OEL) for DEHP (2 mg/m^3^) [50]. The OEL is set at concentrations that, based on available scientific data, should not produce health effects in workers exposed to a substance in the air every day for a workweek throughout their working lifetime (in Switzerland: 8 hours per day, 5 days a week, for 40 years). C_exp_ was calculated based on the following equation:



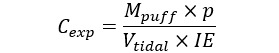



where M_puff_ was the mass of DEHP-d4 in the aerosols generated in a puff by ENDS coupled with the vaping machine (µg per puff), p was the number of puffs per minute that an ENDS user would consume per minute (puff per minute), V_tidal_ was the human mean tidal volume (0.5 L of air per inhalation [51,52]), and IE was the number of inhalations/exhalations per minute.

### Part II: Human Toxicological Study Protocol

#### Participant Sample Size and Selection

A maximum of 10 participants (N=10) will be recruited for repeated exposure to DEHP. Participants who are interested in the study will be checked for eligibility through a short general health questionnaire. These questions are about smoking status, age, BMI, alcohol consumption, general health status, and current job (to exclude the possibility of exposure to phthalates at work). The eligibility criteria are described in Textbox 1.

**Inclusion and exclusion criteria approved by the Ethical Committee. The inclusion criteria “able to understand the procedure” mean the presumed ability of the participants to understand or discern the written and oral explanations during the first visit. This capability will be assessed as follows: by asking the participants to repeat what they understood about the study; by asking questions to the participants and assessing their answers (eg, studied molecules, number of visits, right to accept or reject participation in the study, etc).**

**Inclusion criteria**

- Aged between 18 and 65 years old.
- Men and nonpregnant women (negative pregnancy test).
- Nonsmoker (vaping is authorized).
- Not under a medical regime.
- BMI between 18 and 25.
- Not exposed to phthalates at work.
- Able to understand the procedure.
- Has signed the consent form for research project participation.

**Exclusion criteria**

- Aged 18 years or younger.
- Pregnant women.
- Smoker (cigarette, pipe, and cigar smoker).
- Under a medical regime.
- BMI below 18 or greater than 25.
- Exposed to phthalates at work.
- Not capable of understanding the procedure.
- Has not signed the specific consent form for research project participation.

#### Participant Timeline

**Figure 3:**
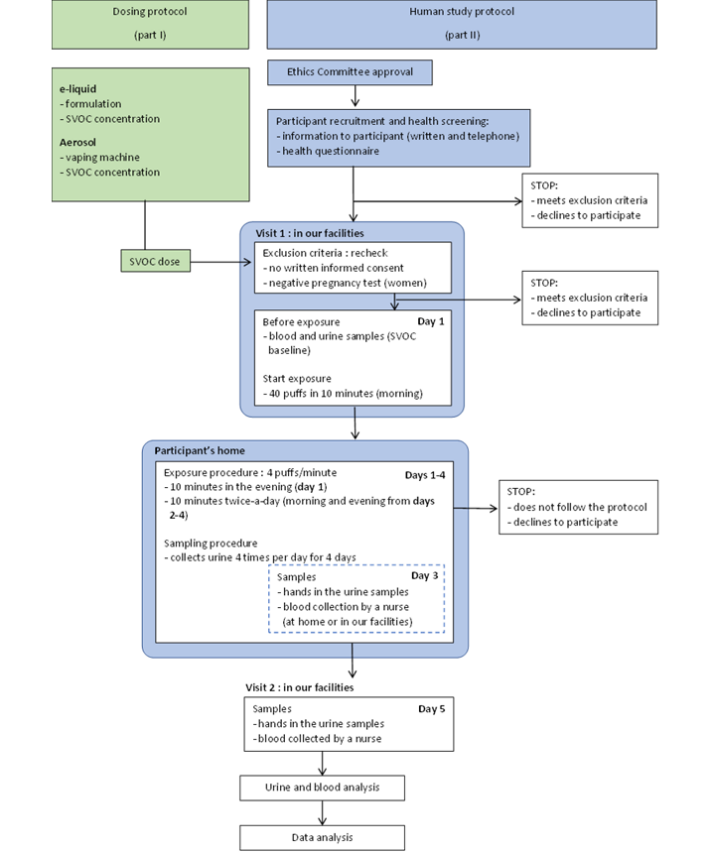
Flowchart of recruitment, screening, visits, interventions, sampling, and analysis. SVOC: semivolatile organic compound.

The flowchart in Figure 3 shows the steps from participant screening and visits through exposure periods until data analysis. Briefly, an initial contact with the participant will be established by email to explain the study and obtain informed consent. Then, a phone call will be made to check if the inclusion and exclusion criteria are met and to get answers for a short general health questionnaire. If the inclusion criteria are met, the participant will be invited for a first visit. At this visit, the participant will sign the written informed consent. Women of reproductive age will also undergo a pregnancy test. Then, the participant will be given an aerosol-forming device and, after being instructed on how to use the device, will undergo the first inhalation exposure under the supervision of a research team member (see “Participant Exposure” section). A blood and urine sample will be collected before the first exposure (baseline) and will serve as baseline concentrations (control). Blood will be drawn again 30 minutes after the first exposure. Then, participants will follow the expected vaping schedule with DEHP-d4-spiked e-liquid and collect spot urine samples 4 times throughout the day (morning, midday, evening, and night) for the 4 days of exposure. Blood and urine collecting times are shown in Multimedia Appendix 1.

#### Blood and Urine Analysis

The Laboratory of Clinical, Forensic, and Environmental Toxicology, University Hospital of Liege, will determine DEHP-d4 and its metabolites concentrations in blood. The quantified metabolites will be deuterated mono(2-ethylhexyl)phthalate (MEHP-d4), deuterated mono(2-ethyl-5-hydroxyhexyl)phthalate (5OH-MEHP-d4), and deuterated mono(2-ethyl-5-oxohexyl)phthalate (5oxo-MEHP-d4). The Institute for Prevention and Occupational Medicine of the German Social Accident Insurance, Institute of the Ruhr-Universität Bochum (IPA), will quantify DEHP-d4 metabolites in urine, as described in Koch et al [53]. The metabolites quantified by the IPA laboratory will be MEHP, 5OH-MEHP, 5oxo-MEHP, and mono(2-ethyl-5-carboxypentyl)phthalate (5cx-MEPP).

#### Participant Exposure

Participants will use the portable aerosol-forming device themselves, first in our facilities (day 1) and then at home (days 1-4). The e-liquid will have a PG/VG ratio of 50/50 with 1% (w/w) ethanol and 0.02% (w/w) DEHP-d4. The participants enrolled in the study will press a button on the ENDS battery. The coil will heat the e-liquid, generating an aerosol with DEHP concentrations less than or equal to the OEL.

The e-liquid will be prepared in advance for DEHP-d4 quantification analysis and will be stored at room temperature during the exposure week. A fraction of the e-liquid will be sent to the analytical laboratory of EPFL to confirm the DEHP-d4 concentration in the e-liquid, and the rest will be frozen at –20 °C until use. On the day of the first exposure to DEHP, the e-liquid will be defrosted. A vial with a weighted amount of e-liquid sufficient for 1-week exposure (ie, reserve) will be prepared for each participant. This vial will be stored at room temperature at the participant’s place, where they will use it to refill the ENDS e-liquid tank when needed. Therefore, a stability test is required to verify DEHP-d4 concentration after several days of storage at room temperature. This step is also important for the relevance of the study. On day 5, the participant will return the ENDS and the e-liquid vial, which will be weighed to calculate the exact amount of e-liquid puffed by the participant, hence the amount of puffed DEHP-d4.

#### Statistical Analysis

Standard descriptive statistics (ie, scatter plots, box plots, means, and SD) will be performed to describe the within-participant evolution of parent compound and metabolite distribution and elimination over time. Absorption and elimination rates will be estimated for each of the participants using standard nonlinear regression methods (hockey plots, piecewise linear regression models, or exponential models). These rates will be compared while accounting for potential effect modifiers (eg, age and gender) using mixed models.

### Ethical Considerations

This study protocol has been approved in Switzerland by the official ethics committee of the Canton de Vaud for research studies with humans (Commission cantonale d'éthique de la recherche sur l'être humain, CER-VD, October 14, 2020, project ID 2020-01095). This study will be carried out at the Department of Occupational and Environmental Health at Unisanté, Lausanne (Switzerland).

This study is in compliance with the Declaration of Helsinki [54], the Swiss legal requirements for scientific research involving human beings [55,56], the Human Research Ordinance (HRO) [57], and the legislation relative to data protection (Swiss Biobanking Platform [SBP]). The Cantonal Ethics Committee of Vaud (CER-VD) approved the first version of the study on October 14, 2020 (project ID 2020-01095). The protocol described in this study is Version 8 of September 14, 2021.

The study is presented to any subjects who contact us for recruitment by phone and through a written participant information sheet. Participants also receive 2 consent forms, one for this study and one for possible future studies. If they agree to participate in the study, they need to sign the informed consent form before being exposed. The information sheet and the consent forms have been approved by the CER-VD.

All health information and biological samples related to this study will be part of a biobank that follows the current Swiss legal requirements for data protection and will be performed according to the Swiss HRO, Article 5. The SBP approved the study on January 21, 2021 (Vita Label N° Unisante_2101_1).

The results from this study will be communicated at scientific meetings and submitted for publication in peer-reviewed journals.

## Results

Here we will present results for the portable aerosol-forming device (Part I) and anticipated results with the participants (Part II).

### Part I: Dosing Protocol

#### E-Liquid Formulation and Testing Conditions Optimization

The optimized e-liquid formulation had a PG/VG ratio of 50/50 with 1% (w/w) ethanol and 0.02% (w/w) DEHP-d4. Ethanol was necessary to dissolve DEHP-d4 and then create a homogeneous solution with PG/VG. The mean recovery in impingers was 89% (SD 5%) of the mass of aerosolized DEHP-d4 (n=5). The optimized number of impingers, impinger volume, and n-hexane volume in each impinger were 3 mL, 125 mL, and 80 mL, respectively. The number of puffs per experiment (n=36) was set to produce 2 dm^3^ of aerosol, a volume that generated quantifiable amounts of DEHP-d4, and that was a submultiple of 1 m^3^ (the volume used to define the OEL), which simplified calculations.

#### DEHP-d4 Mass Balance and Aerosol Concentrations

Total DEHP-d4 mass mean recovery for e-liquids, ENDS heads, and impingers was 100% (SD 22%; n=4). A DEHP-d4 concentration of 0.02% (w/w) in the e-liquid was confirmed by chemical analysis and produced aerosols containing 25.4 (SD 3.4) mg/m^3^ DEHP-d4 (median 26 mg/m^3^), corresponding to a mean of 1.4 (SD 0.2) μg DEHP-d4 per puff (n=6).

#### DEHP-d4 Stability in E-Liquid

**Figure 4:**
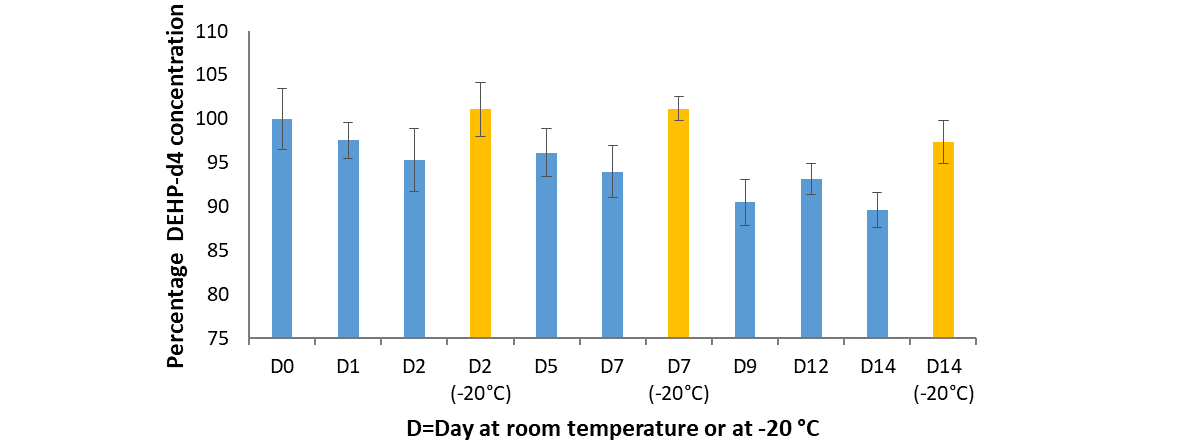
Percentage of isotope-labeled diethylhexyl phthalate (DEHP-d4) concentration over 14 days of storage at room temperature (blue) and at –20 °C (yellow). Note that the y-axis starts at 75%.

The results of the stability test (Figure 4) showed a decrease in DEHP concentration of about 10% when the e-liquid was stored at room temperature. This loss may be explained by slight adsorption of the DEHP to the recipient wall or analytical error (up to 10% is considered in the error range of the analytical method). Therefore, the concentration of DEHP was stable when the e-liquid was stored in the freezer at –20 °C.

#### Aerosol Particle Mass Distribution in E-Liquid

**Figure 5:**
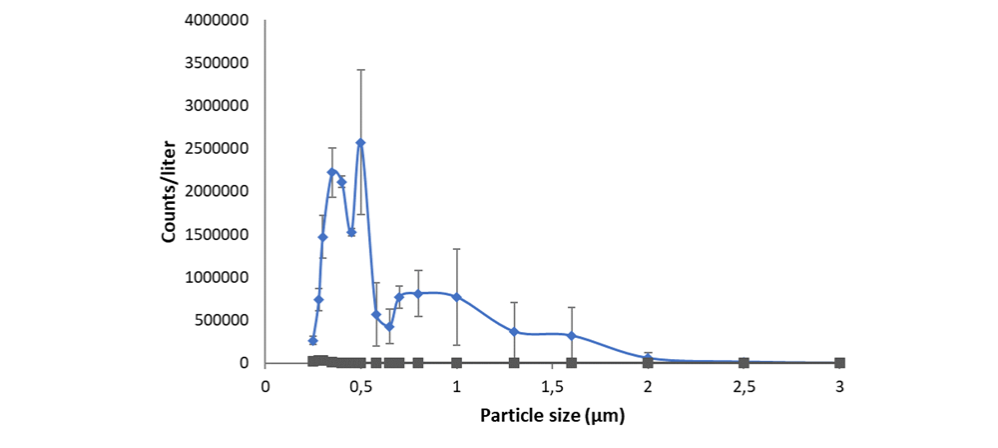
Mean (n=48) particle counts per cubic meter of air measured in ENDS-generated aerosol containing propylene glycol/vegetable glycerol (PG/VG=50/50), 1% ethanol, 0.02% DEHP-d4 (blue line with diamond marks). Gray line and squared marks represent the control (ambient air). Error bars represent the ± SD. Particles with diameters larger than 3 μm were 0.0003% of total counts.

The aerosol particle size distributions are shown in Figure 5. Table 1 lists the particle means expressed in terms of environmental (PM10, PM2.5, and PM1) and occupational (inhalable, thoracic, and respirable) mean mass concentrations. Most of the particles in the diluted ENDS-generated aerosols were at the respirable mass fraction level, with a diameter of 2.5 μm or smaller.

**Table 1:** Mean mass concentrations (μg/m3) corresponding to the aerosol fractions of PM10, PM2.5, and PM1 (PMX: 50% of particles exhibit an aerodynamic diameter below the indicated numbers 10, 2.5, and 1 microns, respectively) as well as the inhalable, thoracic, and respirable fractions (50% of particles exhibit an aerodynamic diameter below the indicated numbers 100, 10, and 4 microns, respectively) as per European Standard EN 481:1993 definitions.

	PM10 (μg/m^3^)	PM2.5 (μg/m^3^)	PM1 (μg/m^3^)	Inhalable (μg/m^3^)	Thoracic (μg/m^3^)	Respirable (μg/m^3^)
Diluted aerosol, mean (SD)	6325 (4363)	6192 (4213)	2634 (728)	6325 (4363)	6325 (4363)	6217 (4245)
Control, mean (SD)	4 (1)	4 (1)	4 (1)	4 (1)	4 (1)	4 (1)

#### DEHP-d4 Exposure Concentration

We observed that on average, an individual puffs 4 times per minute and inhales and exhales 12 times per minute (data not shown). Therefore, a 0.02 % (w/w) DEHP-d4 concentration in the e-liquid generating an aerosol with 1.4 µg per puff of DEHP-d4 led to an estimated exposure concentration C_exp_ of 0.9 mg/m^3^ over a 10-minute exposure, which was below the OEL. Assuming that a participant puffed 40 times on average over the 10-minute exposure duration twice a day (80 puffs per day), the predicted DEHP-d4 dose for a week of exposure was 0.45 mg.

**Figure 6:**
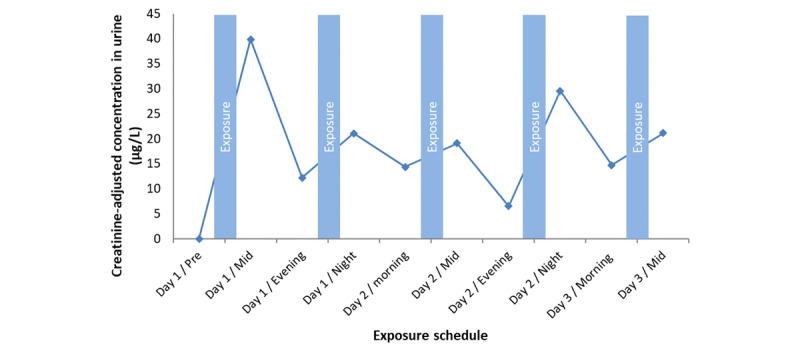
Urinary concentration of the sum of isotope-labeled diethylhexyl phthalate (DEHP-d4) metabolites (MEHP-d4, 5OH-MEHP-d4, 5oxo-MEHP-d4) quantified for 1 participant.

Our first participant could not complete the exposure week because some e-liquid leaked out of the ENDS, preventing our control of the puffed dose. Therefore, results for this participant are shown in Figure 6 as proof of concept. Our results from our first participant showed quantifiable amounts of DEHP-d4 in this participant’s urine. DEHP-d4 was quantified in the e-liquid by chemical analysis before participant exposure (0.019%, w/w). The participant was asked to puff 40 times over 10 minutes twice a day. After five 10-minute exposure periods over 3 days and minor leaking, 2.52 g of e-liquid were used, corresponding to 0.47 mg DEHP-d4. This amount was in line with the DEHP-d4 predicted dose and led to exposure concentrations lower than the OEL but still sufficiently high to quantify DEHP in urine and to elucidate the urinary elimination kinetics. However, C_exp_ led to concentrations below our limit of quantification (LOQ) for blood.

### Part II: Human Toxicological Study Protocol

As of May 2023, we have enrolled 5 participants. Following the whole protocol, participants will be able to generate aerosols with controlled DEHP concentrations using the ENDS and the prepared e-liquid at home for several days.

## Discussion

We developed a method to generate aerosols containing controlled concentrations of SVOCs of toxicological relevance (ie, DEHP in this study) for controlled human exposure studies. Aerosols were generated by a portable and easy-to-use device (ENDS). Our results showed that DEHP in the e-liquid was stable for the duration of the study and that ENDS-generated aerosol particles were in the respirable range. Therefore, DEHP was likely to reach the alveoli, be absorbed, and enter the systemic circulation. Knowledge of human toxicokinetics after SVOC aerosol inhalation exposures will help regulatory agencies set new exposure limits and new requirements for toxicity reporting from manufacturers or suppliers because the data are human data instead of animal data. Some e-liquid leaking occurred with our first participant. This will be prevented by asking the next participants to keep the ENDS at home in a vertical position.

In the near future, our portable aerosol device will be used to study biomarkers of phthalate exposure in humans and the influence of these chemicals on a panel of readouts related to human health. We believe that this protocol with a take-home device has an easy approach, and we therefore anticipate that participant compliance rates to the exposure protocol will be high. The potential limitations of this protocol are the risks of missing part of the dose in blood and urine because only DEHP metabolites, not the parent compound, are quantified in these fluids, and participants are asked for spot urine samples, not 24-hour urine collection for every day of exposure. We hope this protocol will help other researchers design human inhalation studies of environmental substances that can contribute to the large data gap that exists today.

The results of this study will add new and important human data on the toxicokinetics of SVOCs such as DEHP upon repeated, inhalation-only exposures. Understanding phthalate toxicokinetics upon repeated exposure will help researchers understand possible phthalate accumulation in the body (if any) and improve models estimating internal exposures from external exposures, as well as interpret biomonitoring results from the general population, where urine is often collected at different times.

## Appendix

Multimedia Appendix 1 Blood and urine sampling design. The exposure period is shown in grey. On day 1, blood and urine will be taken to provide baseline data (controls). Then, the first exposure starts and will be repeated twice a day, in the morning and in the evening. On day 3, participants return to our facilities to hand out day 1 to day 3 urine samples and for blood sampling. The last exposure is on day 4. On day 5, participants hand out urine samples from day 4 and the morning of day 5; a postexposure blood sample is collected at our facilities.

Multimedia Appendix 2 SCAHT_Experts_Outcome.

## Abbreviations

5cx-MEPP: mono(2-ethyl-5-carboxypentyl)phthalate
5OH-MEHP-d4: mono(2-ethyl-5-hydroxyhexyl)phthalate
5oxo-MEHP-d4: mono(2-ethyl-5-oxohexyl)phthalate
ADME: absorption, distribution, metabolism, and excretion
CER-VD: Cantonal Ethics Committee of Vaud
CORESTA: Cooperation Centre for Scientific Research Relative to Tobacco
CURML: University Centre of Legal Medicine of Lausanne
DEHP: diethylhexyl phthalate
DEHP-d4: isotope-labeled diethylhexyl phthalate
DEP: diethyl phthalate
EDC: endocrine-disrupting chemical
ENDS: electronic nicotine delivery systems
EPFL: Ecole Polytechnique Fédérale of Lausanne
HPLC: high-pressure liquid chromatography
HPVC: high-production-volume chemical
HRO: Human Research Ordinance
IPA: Institute of the Ruhr-Universität Bochum
ISO: International Organization for Standardization
LOQ: limit of quantification
MEHP-d4: mono(2-ethylhexyl)phthalate
OEL: occupational exposure limit
PG: propylene glycol
SBP: Swiss Biobanking Platform
SVOC: semivolatile organic compound
VG: vegetable glycerol
