# Supplementary figures and images for: Repeated Human Exposure to Semivolatile Organic Compounds by Inhalation: Novel Protocol for a Nonrandomized Study
Source: JMIR Res Protoc. 2023 Oct 13;12:e51020. doi: 10.2196/51020 (PMC10612011; doi:10.2196/51020)

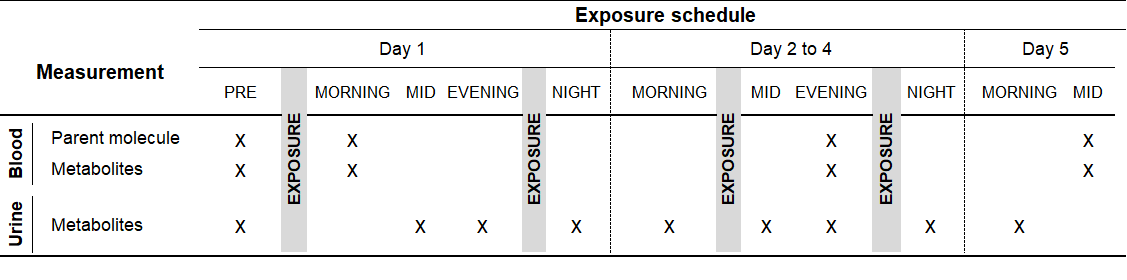

Supplement: Multimedia Appendix 1 [file resprot_v12i1e51020_app1.png]
