# Supplementary material for: Repeated Human Exposure to Semivolatile Organic Compounds by Inhalation: Novel Protocol for a Nonrandomized Study
Source: JMIR Res Protoc. 2023 Oct 13;12:e51020. doi: 10.2196/51020 (PMC10612011; doi:10.2196/51020)
Supplement: Multimedia Appendix 2 [file resprot_v12i1e51020_app2.pdf]

scaht
Swiss Centre for Applied Human Toxicology
Schweizerisches Zentrum für Angewandte Humantoxikologie
Centre Suisse de Toxicologie Humaine Appliquée
Centro Svizzero di Tossicologia Umana Applicata

INFORMATION TO THE APPLICANT
EVALUATION OF ADDITIONAL PROJECT PROPOSALS
SCAHT Additional Projects 2020

Basel, Monday 21 October, 2019

Objective of the call

The SCAHT aims to promote and support cutting-edge research in toxicology relevant to human health protection. As part of the strategic research activities the SCAHT aims to fund a number of short-term projects within the Additional Projects category on an annual basis. For the year 2020 it was decided that thematically, these projects should aim at (in order of priority):

A. Preparation for collaborative projects within the proposed 2021-2024 Research Programme of the SCAHT. Projects should aim at formulating research questions along three principal lines of inquiry:

   1. Investigation of toxicodynamic dose-effect relationships of a selection of (data-poor) compounds of concern to human health. *Output*: Tools (e.g. fit-for-purpose in vitro models, PBTK/IVIVE models) that allow evaluation of biological effects in relation to exposure levels. Better integration of exposure and toxicity by providing an understanding of doses relevant to potential effects on human health.

   2. Advancing the quantitative understanding of existing adverse outcome pathways (AOP). *Output*: Advancement of the AOP-framework (Effectopedia) for future quantitative risk assessment.

   3. Elucidating toxicity pathways and generating data in direct support of AOP development. *Output*: Expansion and development of the qualitative AOP framework through pathway discovery.

B. Supporting or complementing research projects within the Core Domains of the Research Programme 2017 – 2020, however, with research objectives distinct from already on-going projects. The added value to the ongoing projects should clearly be spelled out. An example could be development of a specific technique or method that could be used within current or future Core Projects.

C. Other projects with a focus in line with SCAHT's research objectives will also be considered.

Preference will be given to project proposals in category A, and other proposals will be prioritized in accordance with their ranking in the evaluation. All projects should seek **anchoring in a potential human adverse outcome** related to a disease area.

Regarding the general procedure

- The assessment of scientific excellence was assessed by the SAB. The proposal's relevance and response to the call was assessed internally by the SCAHT.
- The criteria for applicant eligibility are to be evaluated in accordance with the pre-defined criteria as decided upon by the SCAHT Management Board and all applicants receive equal treatment regardless of any personal aspects.
- An overall ranking of the project proposals was created by taking into account the SAB evaluation, the participants' scientific productivity and contribution to SCAHT objectives other than research, as well as the recommendation of the Management Board.
- All information and documents transferred by applicants to the SCAHT were treated as confidential.

1. Evaluation criteria for contribution to SCAHT objectives (SCAHT Directorate's evaluation)

The following aspects of the project proposal was regarded and scored according to the tables (below):

- ***Response to the call:*** How well does the proposal respond to the call and/or the SCAHT research strategy?

Description	Criteria	Score
Response to categories A, B or C, of the call.	Not responding	1
	Category C	2
	Category B	3
	Category A	5

- ***Scientific output:*** H-index of the principal investigator of the project proposal.

Description	Criteria	Score
H-index	<10	1
	10-20	2
	20-30	3
	30-40	4
	>40	5

- ***Strengthening of the SCAHT network:*** Scoring the number of collaborating groups (internal and external to SCAHT) participating to the proposed project.

Description	Criteria	Score
Number of Collaborating groups	No groups	1
	1 group	2
	2 groups	3
	3 groups	4
	4 or more	5

- ***Contribution to teaching***: do project applicants contribute to teaching in the MAS in Toxicology, or to the MSc in Drug Sciences?

Description	Criteria	Score
Applicants actively participating in teaching duties	Not contributing	1
	Contributing	5

- ***Contribution to the regulatory community:*** do applicants contribute to the regulatory toxicology field? Through what type of activity?

Description	Criteria	Score
Activity of regulatory interest [$]	None	1
	Participant in expert committee	2
	Review of test guideline/AOP	3
	AOP development	5

[$] The score here is based on an estimation on the time-consumption of the activity, AOP development being by far the most time consuming of the listed (thus highest score)

Lowest total score is 5, and maximal is 25

2. The evaluation criteria for scientific excellence by the SAB

The following aspects of the project proposal was regarded and a **short** comment to each category was given in the table below:

- ***Clarity of the Proposal***: is the proposal clear and well drafted? Are objectives, milestones and deliverables well defined and appropriate?

- ***Scientific relevance*** and originality: would the outcome of the project advance knowledge in its domain, or in human toxicology?

- ***Overall probability of success***: quality and feasibility of the proposed plans, looking at goals and objectives, research focus and themes, feasibility of the proposed approach and research methods.

- ***Added value*** of the outcome of proposed project; either through advancing research in its domain, or by supporting regulatory sciences.

The SAB was asked to rank the project proposals in order of priority for funding, where high=20p, medium=10p and low=5p.

scaht
Swiss Centre for Applied Human Toxicology
Schweizerisches Zentrum für Angewandte Humantoxikologie
Centre Suisse de Toxicologie Humaine Appliquée
Centro Svizzero di Tossicologia Umana Applicata

Evaluation Tables

Table 1 – SCAHT Directorate evaluation of Project Proposals 2021-2024

Proposal N°	Title	Project lead	Budget ask	Response to the call	Scientific output	Strengthening of the SCAHT network	Contribution to teaching	Contribution to the regulatory community	*Total Score*
**P6**	Inhaled phthalate aerosols and effects on semen quality – a feasibility study	Myriam Borgatta	60'000	2	1	2	5	1	**11**
Note:					H=3				

Table 2 – SAB rank of project proposals AP2020

Reviewer	Title	Project lead	Clarity of proposal	Scientific relevance	Overall probability of success	Added value	*Rank*
**1**	Inhaled phthalate aerosols and effects on semen quality – a feasibility study	Myriam Borgatta	Very clear application, Well aligned to call text, This is an A-project.	Development of much needed methodology to determine human EDC exposure via inhalation in work environment. The project also includes effect measurements (semen parameters).	Very high. Pilot project to support proposed SCHAT core project for the period 2021/24.	Will allow for improved human EDC monitoring. Well connected to SCHAT Theme EDCs and male reproduction	High
Note:							
**2**	Inhaled phthalate aerosols and effects on semen quality – a feasibility study	Myriam Borgatta	Clear, but power analysis missing	This is a real human toxicology study, and a direct and bold approach	Ethics approval? Power analysis. It is not sure whether n=5 is sufficient, this should be checked beforehand	Link to EDC projects. Human PK data useful as experience for other projects	High
Note:							

Dear SCAHT,

About the "SCAHT additional projects" of 2020, we are pleased to submit a revised proposal with the reduced budget.

The most important step of this project is to control the exposure before exposing any participant. Therefore, the two first aims from the original project were kept, which are (1) to adapt a delivery system and (2) establish a protocol for chronic exposure, including a questionnaire for participants. The sperm quality assessment is no longer part of this additional project. We are now focusing solely on toxicokinetics/dynamics of phthalates.

The work percentage of the mechanical engineer was adapted to compensate the actual time needed in the project.

Please, do not hesitate to contact me if need be.

Best regards,
Myriam

Dr. ès Sc. Myriam Borgatta | Responsable de recherche

unisanté
Centre universitaire
de médecine générale
et santé publique • Lausanne
Département Santé au travail et environnement
Route de la Corniche 2
1066 Epalinges ▪ Suisse

Depuis le 1er janvier 2019, la Policlinique médicale universitaire, l'Institut universitaire de médecine sociale et préventive, l'Institut universitaire romand de santé au travail et l'association Promotion Santé Vaud, forment unisanté, Centre universitaire de médecine générale et santé publique à Lausanne. Voir le lien suivant : www.unisante.ch
